# Risk factors for severe COVID-19 in the young—before and after ICU admission

**DOI:** 10.1186/s13613-023-01127-8

**Published:** 2023-04-25

**Authors:** Johanna Kämpe, Olof Bohlin, Martin Jonsson, Robin Hofmann, Jacob Hollenberg, Rebecka Rubenson Wahlin, Per Svensson, Per Nordberg

**Affiliations:** 1 Department of Clinical Science and Education, Södersjukhuset, Centre for Resuscitation Science, Karolinska Institutet, 11883 Stockholm, Sweden; 2 Department of Clinical Science and Education, Södersjukhuset, Karolinska Institutet, 11883 Stockholm, Sweden; 3 Function Perioperative Medicine and Intensive Care, Karolinska University Hospital, 17177 Stockholm, Sweden

## Abstract

**Background:**

Factors associated with severe COVID-19 and death among young adults are not fully understood, including differences between the sexes. The aim of this study was to identify factors associated with severe COVID-19 requiring intensive care and 90-day mortality among women and men below 50 years of age.

**Methods:**

A register-based study using data from mandatory national registers, where patients with severe COVID-19 admitted to the ICU with need for mechanical ventilation (cases) between March 2020 and June 2021 were matched regarding age, sex, and district of residence with 10 population-based controls. Both the study population and the controls were divided into groups based on age (< 50 years, 50–64, and ≥ 65 years) and sex. Multivariate logistic regression models including socioeconomic factors were used to calculate odds ratios (OR) with 95% confidence intervals (CIs) for associations between severe COVID-19 in the population to compare the magnitude of the risk associations for co-morbidities in the different age categories, and subsequently factors associated with 90-day mortality among patients admitted to ICU.

**Results:**

In total, 4921 cases and 49,210 controls (median age 63 years, 71% men) were included.

The co-morbidities with the strongest associations with severe COVID-19 for the young population compared to older patients were chronic kidney disease (OR 6.80 [3.61–12.83]), type 2 diabetes (OR 6.31 [4.48–8.88]), hypertension (OR 5.09 [3.79–6.84]), rheumatoid arthritis (OR 4.76 [2.29–9.89]), obesity (OR 3.76 [2.88–4.92]), heart failure (OR 3.06 [1.36–6.89]), and asthma (OR 3.04 [2.22–4.16]).

When comparing women vs. men < 50 years of age, stronger associations were seen for women regarding type 2 diabetes (OR 11.25 [6.00–21.08] vs OR 4.97 [3.25–7.60]) and hypertension (OR 8.76 [5.10–15.01] vs OR 4.09 [2.86–5.86]).

The factors associated with 90-day mortality in the young were previous venous thromboembolism (OR 5.50 [2.13–14.22]), chronic kidney disease (OR 4.40 [1.64–11.78]) and type 2 diabetes (OR 2.71 [1.39–5.29]). These associations with 90-day mortality were foremost driven by the female population.

**Conclusion:**

Chronic kidney failure, type 2 diabetes, hypertension, rheumatoid arthritis, obesity, heart failure, and asthma were the strongest risk factors associated with severe COVID-19 requiring ICU-care in individuals < 50 years compared to the older population. However, after ICU admission, previous thromboembolism, chronic kidney failure, and type 2 diabetes were associated with increased 90-day mortality. The risk associations for co-morbidities were generally stronger among younger individuals compared to older and in women compared to men.

**Supplementary Information:**

The online version contains supplementary material available at 10.1186/s13613-023-01127-8.

## Introduction

High age and male sex have been shown to be the predominant risk factors for developing severe COVID-19 [1, 2]. However, a substantial number of younger patients have also been severely affected with need for mechanical ventilation at the intensive care unit, and ultimately, at risk for a fatal outcome. This group may be of particular interest since they most often do not consider themselves at risk for developing severe disease and, in relation to the COVID-19 pandemic, this group has been shown to be less willing to follow behavioral restrictions and recommended vaccination programs [3].

Observational data show both higher prevalence of COVID-19 and a more severe disease course among individuals with cardiometabolic disease [1, 2, 4–11], respiratory disease, chronic kidney disease, and systemic inflammatory diseases [1]. Socioeconomic factors are independent risk factors for acquiring severe COVID-19 but are also strongly related to the metabolic syndrome [12] and when investigating the impact of cardiometabolic co-morbidities this needs to be accounted for.

In this nationwide study, we aimed to study associations between co-morbidities and risk for acquiring severe COVID-19 (defined as the need for mechanical ventilation at the ICU) among younger (< 50 years of age) men and women as well as factors associated with 90-day mortality. Because male sex is a strong separate risk factor for severe COVID-19, we stratified the analyses on sex to better estimate the co-morbidities differential contribution to disease severity.

## Methods

We performed a nationwide case–control study using data from the Swedish Intensive Care Registry (SIR) [13], where a total of 4921 patients with severe COVID-19 (cases) from March 2020 to June 2021 were matched with 10 population-based controls each (in total 49 210 controls). In addition, the cohort of all cases were used to calculate 90-day mortality. The study was approved by the Swedish Ethical Review Authority (DNR2020/124-31/4).

### Patients

Cases were defined as all patients (≥ 18 years of age) admitted to Swedish intensive care units (ICU), in need of invasive mechanical ventilation (IMV), with a PCR confirmed COVID-19 infection reported to SIR between March 2020 and June 2021. In this study it was not possible to compare our cases with COVID-19 patients admitted to hospital but not in need of intensive care. Therefore, to assess the association between risk factors and severe COVID-19, cases were compared to matched controls drawn from the general population. For each case, 10 population-based controls were randomly selected from the Swedish Population Register and matched regarding age, sex, and district of residence [14]. The controls had not been treated for COVID-19 at any Swedish ICU facility.

The study population was divided into six groups based on age (< 50 years, 50–64, and ≥ 65 years) and sex, and then compared with matched control subjects.

When studying co-morbidities affecting 90-day mortality, only the patients admitted to ICU were considered.

### Data collection and registries

For patient identification and as primary data source SIR was used. This national register with > 95% coverage of all ICU admissions [13] provided date of admission, need for IMV, length of stay at the ICU, complications and other ICU measures. The study database was merged with multiple Swedish national registries (Additional file 1) using everyone’s unique personal identification number [15].

The Swedish Longitudinal Integrated Database for Health Insurance and Labour Market Studies (LISA) [14] includes data on socioeconomic variables and sociodemographic variables, including district of residence, region of birth, level of education and income status.

Data on medical diagnosis according to the International Classification of Diseases version 10 (ICD-10), for both cases and control subjects were collected from the National Patient Registry (NPR) [16] which also provides data on inpatient care in Sweden. Data on dispensed prescribed drugs were collected from the Swedish Prescribed Drugs Register. This registry contains information about the dispensed prescribed drugs according to the Anatomical Therapeutic Chemical Classification (ATC-code).

### Exposures

Exposures were defined as a medical history of both cardiometabolic disease and other conditions, classified using the ICD-10 (Additional file 1).

Diagnoses were based on primary and secondary diagnoses in the National Patient Register within 15 years preceding ICU admission, and prescribed drugs within the preceding 12 months. Diagnoses were defined as being pre-existing if they appeared in medical registries earlier than 30 days before ICU admission, or/and if prescribed medications for a specific diagnosis were prescribed and collected from the pharmacy before admission.

### Definition of covariates and variables for analyses

Level of education was defined by the highest completed level of education and categorized into primary education (≤ 9 years), secondary education (10–12 years) and post-secondary education (> 12 years). Region of birth was defined as: (1) being born in Sweden, (2) a Nordic country (excluding Sweden), (3) European Union 15 (17), (4) another EU country, (5) Asia, (6) Africa, (7) South America, or (8) other region of birth (North America, Oceania, and former Soviet Union). Marital status was defined as either married or not married. Region of birth, sex (male/female), and age constituted variables for subgroup analyses. The income variable was categorized into three levels and defined based on the household’s disposable income in the past year.

### Outcome

Primary outcome was defined as an ICU admission due to COVID-19 (with a laboratory-confirmed SARS-CoV-2-infection), registered in SIR, with at least one episode of IMV during the ICU stay and defined all cases in the case–control design.

In the ICU cohort, including all cases, we performed a secondary analysis where outcome was defined as death at 90 days after ICU-admission. 90-day mortality was calculated from both SIR (death at ICU) and using the Swedish Cause of Death Registry to include those who died after discharge from the ICU. For the analysis on mortality, we added body mass index (BMI) above 30, to define obesity together with the ICD code.

Because of reduced power in assessing associations between co-morbidities and 90-day mortality in the young cohort, only the co-morbidities that were associated with severe COVID-19 below a Bonferroni corrected significance level (*p* < 0.05/16) were considered.

### Statistical methods

Categorical variables are reported as frequencies and percentages, continuous variables are reported as median and interquartile range (IQR) or as mean and standard deviation (SD) as appropriate. Co-morbidities preceding SARS-CoV-2 infection and their association with severe COVID-19, defined as admitted to ICU with need of mechanical ventilation, were assessed using a conditional logistic regression model adjusted for age, sex, sociodemographic and socioeconomic variables (marital status, region of birth, disposable income, and educational level). For each ICU-case, 10 population-based controls matched for age, sex, and district of residence were randomly drawn from LISA (14), and information about their co-morbidities were taken from NPR [16]. None of the population based-controls had been admitted to an ICU due to COVID-19.

Association between preceding co-morbidities and 90-day mortality (within cohort analysis, only among cases admitted to ICU) were calculated using a standard logistic regression model adjusted for age and sex. Results are presented as odds ratio (OR) with 95% confidence interval (CI). We considered a two-sided *p-*value < 0.05 statistically significant and corrections for multiplicity were made. All statistical analyses were performed using R (version 4.1.1).

## Results

During the study period, 10,212 ICU admissions due to severe COVID-19 were registered, including 7435 unique patients, of which 4921 required IMV at some point of their ICU stay (cases). For each case 10 matched control subjects were selected from the general population, in total 49,210 control subjects. The selection of the study population and reasons for exclusion are described in (Additional file 1).

### Baseline characteristics

In the whole cohort of ICU patients, the median age was 63 years (IQR 54–72) and 71% were men. The younger group of patients, < 50 years of age, consisted of 817 patients and 66% men. Baseline characteristics of all cases and controls are summarized in (Table 1). Cases were less likely to have post-secondary education, lower income and were to a larger extent born outside Sweden compared to the controls. Co-morbidities were in general more common in cases compared to controls, most prominently, hypertension, type 2 diabetes, and obesity.

**Table 1:** General characteristics of cases and population-based controls

	Cases	Controls
Number (*n*)	4921	49 210
Age (mean (SD))	62.45 (13.00)	62.45 (13.00)
Age group (%)		
Less than 50	817 (16.6)	8580 (17.4)
Between 50 and 64	1797 (36.5)	18 120 (36.8)
More than 65	2307 (46.9)	22 510 (45.7)
Sex (women (%))	1447 (29.4)	14 470 (29.4)
Income category (%)		
1	2146 (43.7)	15 863 (32.3)
2	1526 (31.1)	16 483 (33.6)
3	1237 (25.2)	16 772 (34.1)
Education (%)		
Primary	1385 (29.1)	10 216 (21.1)
Secondary	2141 (45.0)	22 023 (45.5)
Post-secondary	1236 (26.0)	16 196 (33.4)
Region of birth (%)		
Africa	213 (4.3)	1199 (2.4)
Asia	861 (17.5)	4286 (8.7)
EU15, excl. Nordic	98 (2.0)	842 (1.7)
Europa, excl. EU15 and Nordic	436 (8.9)	3250 (6.6)
North America	19 (0.4)	176 (0.4)
Nordic countries, excl. Sweden	202 (4.1)	1669 (3.4)
Oceania	1 (0.0)	11 (0.0)
Unknown	5 (0.0)	8 (0.0)
Soviet Union	3 (0.1)	50 (0.1)
Sweden	2 989 (60.7)	37 224 (75.6)
South America	94 (1.9)	495 (1.0)
Pre-existing diagnoses (%)		
Hypertension	1753 (35.6)	11 524 (23.4)
Kidney disease	242 (4.9)	781 (1.6)
Rheumatoid arthritis	132 (2.7)	604 (1.2)
Systemic inflammatory disease	136 (2.8)	718 (1.5)
Inflammatory bowel disease	81 (1.6)	658 (1.3)
Venous thromboembolism	270 (5.5)	1570 (3.2)
Obesity	529 (10.7)	1770 (3.6)
Chronic obstructive pulmonary disease	231 (4.7)	1273 (2.6)
Myocardial infarction	275 (5.6)	1871 (3.8)
Heart failure	296 (6.0)	1635 (3.3)
Atrial fibrillation	426 (8.7)	3222 (6.5)
Type 1 diabetes	202 (4.1)	964 (2.0)
Asthma	388 (7.9)	1799 (3.7)
Type 2 diabetes	948 (19.3)	4223 (8.6)
Angina	284 (5.8)	1973 (4.0)
Malignancy	431 (8.8)	4142 (8.4)

### Comparison of risk factors between patients and controls

**Fig. 1:**
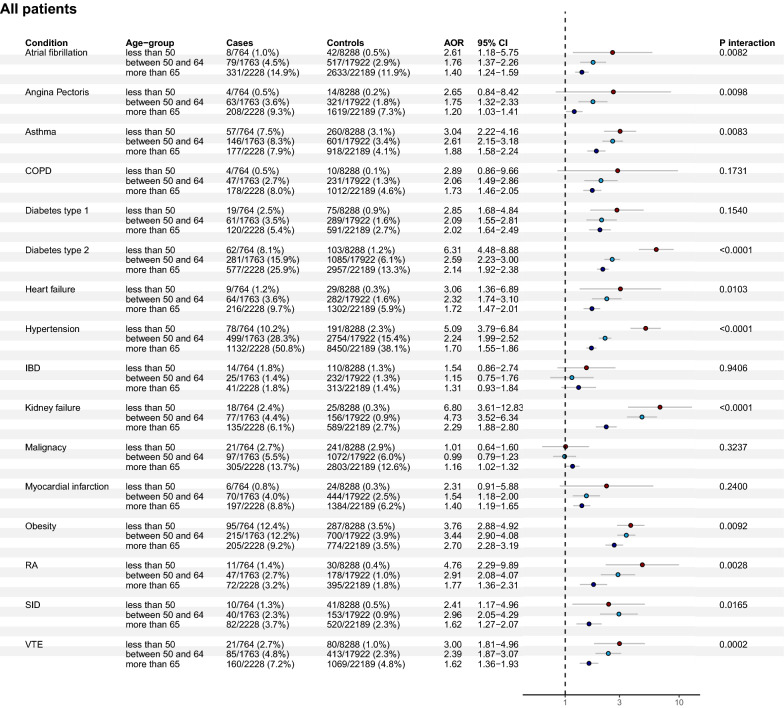
Associations of pre-existing co-morbidities and severe COVID-19 (adjusted ORs with 95% CIs and p-value for age interaction) in all three age groups. This fully adjusted model included co-variates of age, sex, sociodemographic and socioeconomic variables (marital status, region of birth, disposable income, and educational level) for all exposures

**Fig. 2:**
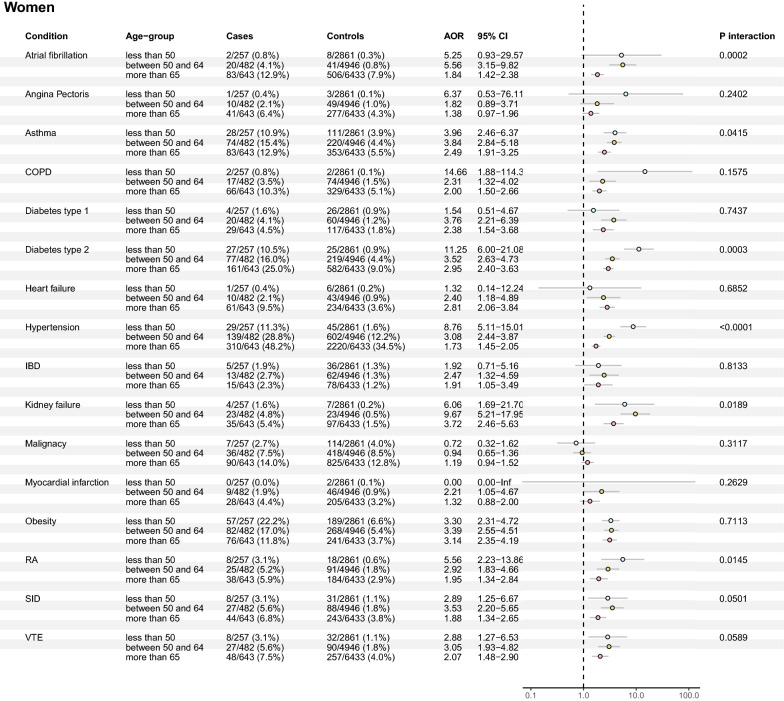
Associations of pre-existing co-morbidities and severe COVID-19 (adjusted ORs with 95% CIs, and p-value for age interaction) in all three age groups of women. This fully adjusted model included co-variates of age, sociodemographic and socioeconomic variables (marital status, region of birth, disposable income, and educational level) for all exposures

**Fig. 3:**
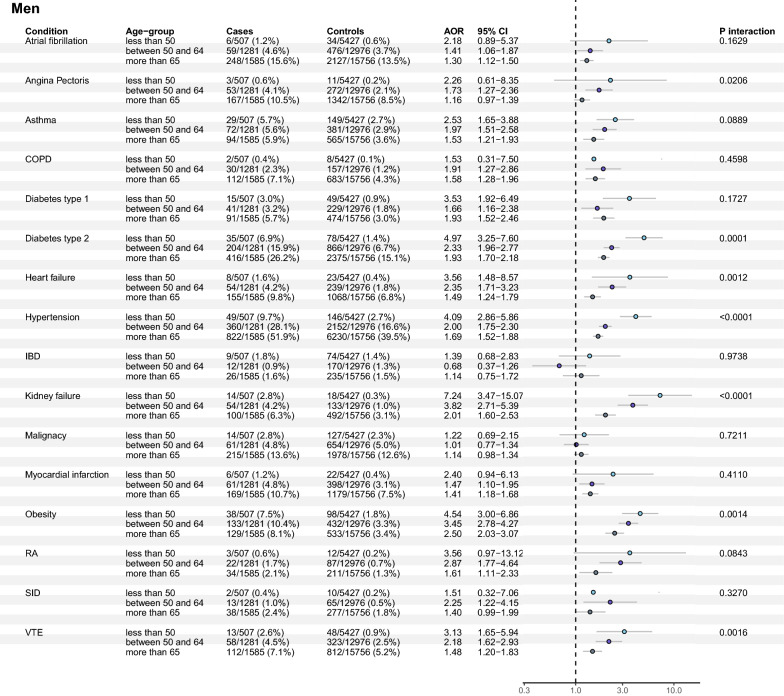
Associations of pre-existing co-morbidities and severe COVID-19 (adjusted ORs with 95% CIs, and p-value for age interaction) in all three age groups of men. This fully adjusted model included co-variates of age, sociodemographic and socioeconomic variables (marital status, region of birth, disposable income, and educational level) for all exposures

The results of the multivariate logistic regression analysis of risk factors for severe COVID-19, requiring ICU admission and IMV, are presented for all cases, and for women and men separately in (Figs. 1, 2, 3).

**Fig. 4:**
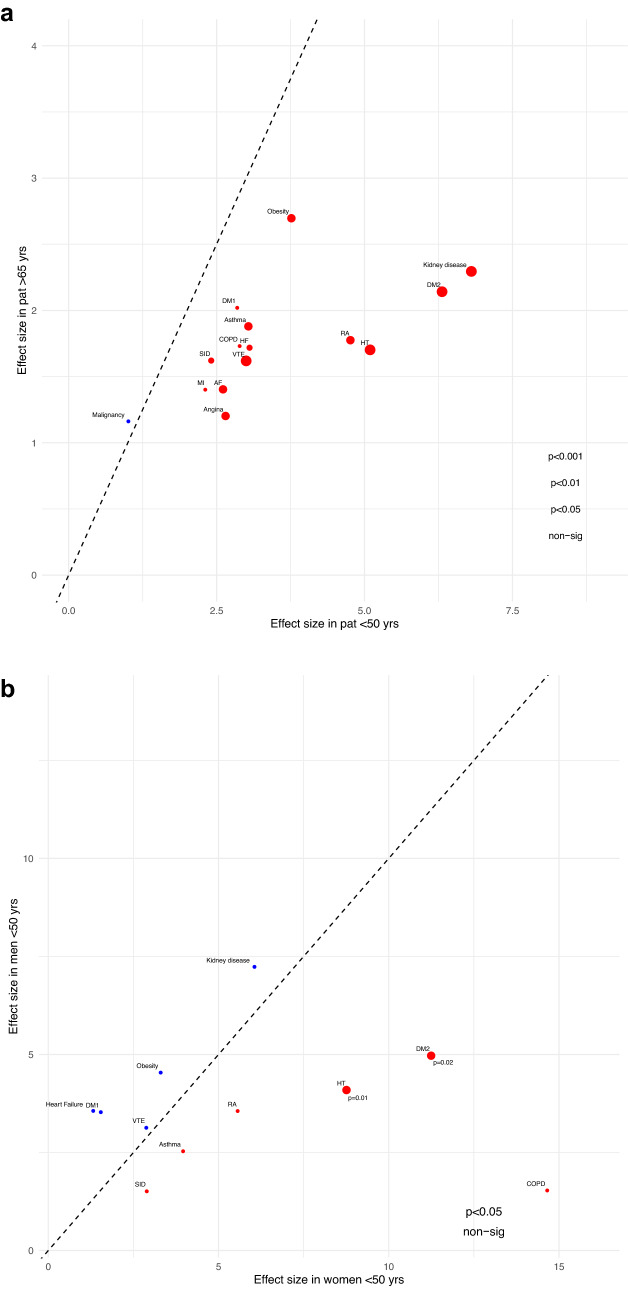
**a***.* *Co-morbidites and severe COVID-19.* Comparison of effect sizes between young (< 50 years) and old individuals (> 65 years). As shown, effect sizes for the co-morbidites are generally larger in the younger population. Co-morbidities without evidence of association (p > 0.05) in any group are not shown. [Adjusted ORs, 95% CIs, p-values for significant age interactions are indicated by the circle size for each condition. **b**: *Co-morbidites and severe COVID-19.* Comparison of effect sizes between women and men in the young age group (< 50 years). Hypertension and type 2 diabetes display significantly larger effect sizes in women compared to men. Co-morbidities without evidence of association (*p* > 0.05) in any group are not shown. [Adjusted ORs, 95% CIs, and p-values for significant sex interactions are indicated by the circle size for each condition]

The co-morbidities that displayed the strongest associations with severe COVID-19 in young individuals were: chronic kidney disease (OR 6.80 [3.61–12.83], type 2 diabetes (OR 6.31 [4.48–8.88]), hypertension (OR 5.09 [3.79–6.84]), rheumatoid arthritis (OR 4.76 [2.29–9.89]), obesity (OR 3.76 [2.88–4.92]), heart failure (OR 3.06 [1.36–6.89]), and asthma (OR 3.04 [2.22–4.16]). Notably, in patients below 50 years of age the co-morbidities associated with severe COVID-19 frequently displayed a larger effect size compared to older age groups (Fig. 4a).

When comparing women and men in the younger age group (< 50 years of age), women had higher associated risks related to type 2 diabetes (OR 11.25 [6.00–21.08] vs OR 4.97 [3.25–7.60], *p* = 0.02 (sex interaction)) and hypertension (OR 8.76 [5.11–15.01] vs OR 4.09 [2.86–5.86], *p* = 0.01 (sex interaction)). None of the co-morbidities had a significantly larger effect size in men (Fig. 4b).

### Disease severity within ICU cases

In our cohort of patients below 50 years of age the 90-day overall mortality was 8,7% whereas in patients above 50 years of age the overall mortality rate was 35,4%. For the young age group, the 90-day mortality rate did not differ between women and men (8,9% and 8,5% respectively, *p* = 0.73). Baseline characteristics of all ICU patients per age groups are presented in (Table 2).

**Table 2:** General characteristics of cases at ICU admission

	< 50 years	50–64 years	> 65 years
Number (*n*)	817	1797	2307
Age (mean (SD))	40.21 (7.85)	57.77 (4.23)	72.76 (5.27)
Sex (women (%))	281 (34.4)	496 (27.6)	670 (29.0)
Income category (%)			
1	455 (56.0)	690 (38.5)	1001 (43.5)
2	251 (30.9)	584 (32.6)	691 (30.0)
3	106 (13.1)	520 (29.0)	611 (26.5)
Education (%)			
Primary	178 (23.2)	406 (23.0)	801 (36.0)
Secondary	369 (48.2)	852 (48.2)	920 (41.3)
Post-secondary	219 (28.6)	510 (28.8)	507 (22.8)
Region of birth (%)			
Africa	64 (7.8)	86 (4.8)	63 (2.7)
Asia	222 (27.2)	383 (21.3)	256 (11.1)
EU15, excl. Nordic	5 (0.6)	28 (1.6)	65 (2.8)
Europa, excl. EU15 and Nordic	78 (9.5)	160 (8.9)	198 (8.6)
North America	2 (0.2)	12 (0.7)	5 (0.2)
Nordic countries, excl. Sweden	4 (0.5)	73 (4.1)	125 (5.4)
Oceania	0 (0.0)	1 (0.1)	0 (0.0)
Soviet Union	0 (0.0)	1 (0.1)	2 (0.1)
Sweden	425 (52.0)	1 016 (56.5)	1 548 (67.1)
South America	16 (2.0)	34 (1.9)	44 (1.9)
Unknown	1 (0.1)	3 (0.2)	1 (0)
Pre-existing diagnoses (%)			
Hypertension	84 (10.3)	507 (28.2)	1162 (50.4)
Kidney disease	21 (2.6)	79 (4.4)	142 (6.2)
Rheumatoid arthritis	11 (1.3)	49 (2.7)	72 (3.1)
Systemic inflammatory disease	11 (1.3)	42 (2.3)	83 (3.6)
Inflammatory bowel disease	14 (1.7)	25 (1.4)	42 (1.8)
Venous thromboembolism	21 (2.6)	85 (4.7)	164 (7.1)
Obesity	262 (32.1)	515 (28.7)	467 (20.2)
Chronic obstructive pulmonary disease	4 (0.5)	47 (2.6)	180 (7.8)
Myocardial infarction	6 (0.7)	71 (4.0)	198 (8.6)
Heart failure	9 (1.1)	65 (3.6)	222 (9.6)
Atrial fibrillation	8 (1.0)	81 (4.5)	337 (14.6)
Type 1 diabetes	19 (2.3)	62 (3.5)	121 (5.2)
Asthma	58 (7.1)	149 (8.3)	181 (7.8)
Type 2 diabetes	69 (8.4)	287 (16.0)	592 (25.7)
Angina	4 (0.5)	65 (3.6)	215 (9.3)
Malignancy	22 (2.7)	97 (5.4)	312 (13.5)

Diagnoses are defined as pre-existing if appearing > 30 days before ICU-admission

**Fig. 5:**
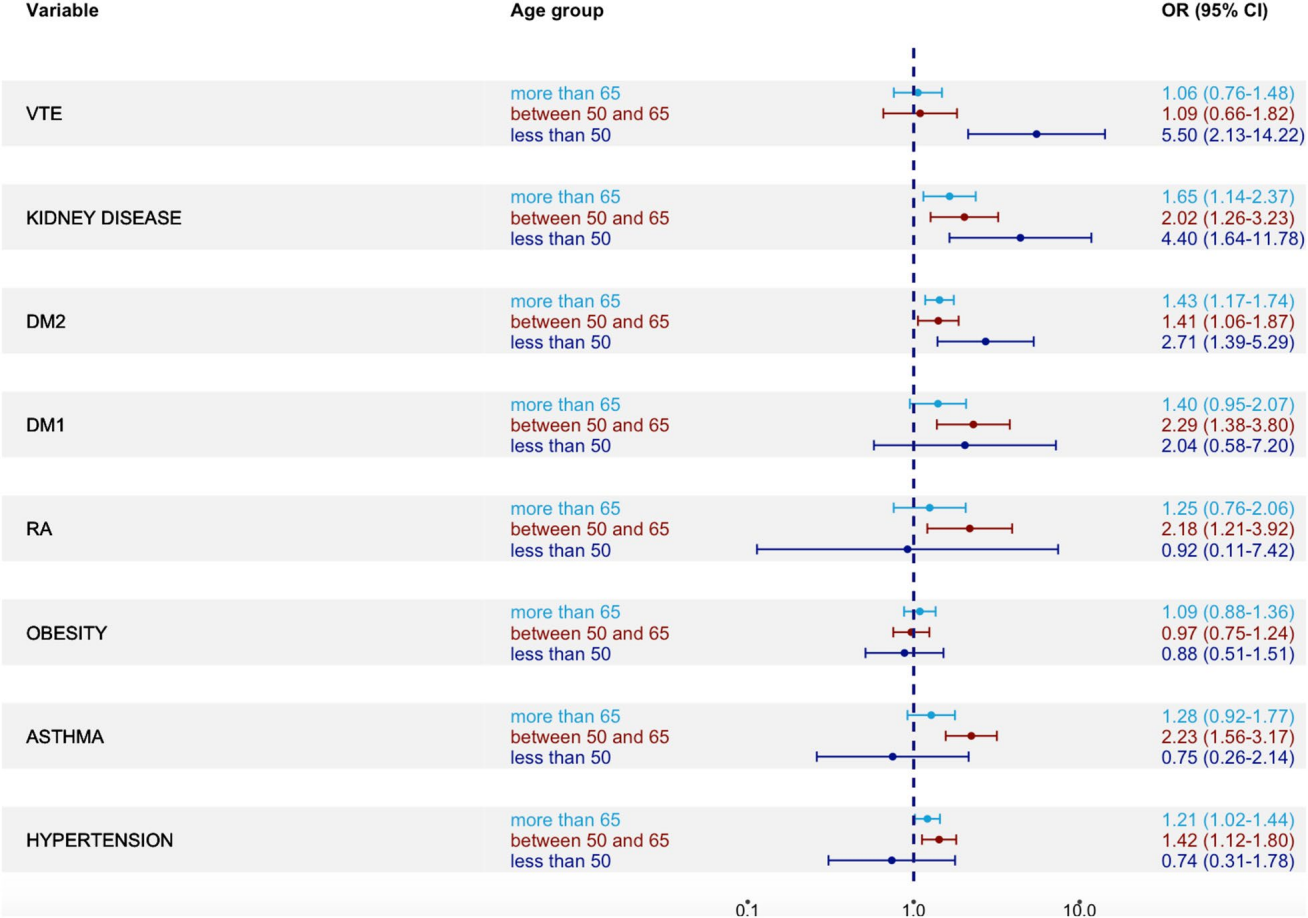
Associations of co-morbidities and 90-day mortality (adjusted ORs with 95% CIs) in all three age groups. This adjusted model included co-variates of age and sex

In (Fig. 5) associations between co-morbidities and 90-day mortality are shown for all three age categories. For the young age group co-morbidities significantly associated with 90-day mortality were: previous venous thromboembolism (OR 5.50 [2.13–14.22], *p* = 0.0004), chronic kidney disease (OR 4.40 [1.64–11.78], *p* = 0.0032), and type 2 diabetes (OR 2.71 [1.39–5.29], *p* = 0.0034). There was a general tendency that the associations in the young cohort were driven by the female population. In the sex stratified analysis, only previous venous thromboembolism was significantly associated with 90-day mortality in both sexes. In contrast, chronic kidney disease (OR 12.4 [2.3–68.4] vs OR 2.8 [0.76–10.4]), and type 2 diabetes (OR 3.9 [1.4–10.3] vs OR 2.10 [0.82–5.36]) only showed a significant association in women [Additional file 1].

## Discussion

In this nationwide study on associations between co-morbidities and risk for acquiring severe COVID-19 among young men and women, our main findings were that certain risk factors, such as chronic kidney disease, type 2 diabetes, hypertension, rheumatoid arthritis, obesity, heart failure, and asthma were more important for the development of severe COVID-19 among young individuals compared to older aged individuals. In the young age group, we also observed a clear sex difference, where type 2 diabetes and hypertension displayed a significantly larger impact for the developing severe COVID-19 in women compared to men. After admission to the ICU, previous venous thromboembolism, chronic kidney disease and type 2 diabetes were associated with increased 90-day mortality in the young age group, with a stronger relative effect in women (Additional file 1). Considering that younger individuals might not consider themselves at risk for developing severe disease and are less willing to follow governmental restrictions and recommended vaccination programs [3], these results, predominately based on data from unvaccinated patients, may have several important implications for future educational and preventive efforts.

Many factors such as frailty or co-morbidities on top of the primary diagnosis (i.e., COVID-19) contribute to the necessity of ICU admission. In our cohort, older individuals (> 65 years) constitute nearly half of all ICU admissions. As expected, our study supports previous findings that there is a certain risk for severe COVID-19 related to the high age. Among young individuals (< 50 years of age) it appears that chronic kidney disease, type 2 diabetes, hypertension, and obesity all substantially impact the risk of developing severe COVID-19. A majority of these co-morbidities are part of the metabolic syndrome [5], highly correlated in our cohort (Additional file 1), and previously known to impact the severity of COVID-19 [11]. We also found clear sex differences in younger individuals, where type 2 diabetes and hypertension presented significantly higher effect sizes in women compared to men while none of the co-morbidities had a significantly higher effect size in men. It is known that men are naturally more at risk for severe COVID-19 infection and death [18], and this is supported by our finding since men were largely overrepresented in our ICU-cohort (nearly twice as many compared to women). Therefore, it may be expected that women need to have additional risk factors to become severely ill in COVID-19, which is in line with our results.

Assessing mortality, the most severe effect of COVID-19 infection, younger individuals were less likely to die (8,7% in patients < 50 years vs 35,4% in patients > 50 years. We observed strong associations with 90-day mortality and previous venous thromboembolism, chronic kidney disease, and type 2 diabetes in patients less than 50 years of age even though the absolute mortality risk was lower in the younger individuals. However, the mortality risk seen in the whole group of younger individuals tended to be driven by the group of young females. Still, this does not imply that the absolute mortality risk was higher in women. In our cohort, the 90-day mortality rate did not differ among sexes in the young. Our results show that even though men in general are more likely to become severely ill due to COVID-19, women demonstrate the same absolute mortality risk as men once in need of IMV in the ICU.

Despite available vaccines since late 2020, as of December 2022, 73% [19] of the Swedish population had received three doses of vaccine and were adequately protected against severe disease. For comparison, 73% of the European population and 69% of the population in the USA were fully vaccinated in June 2022 [20]. Hence, there is still a significant number of individuals that are not fully vaccinated against COVID-19 and foremost younger individuals are unvaccinated (in Sweden 16.4% < 50 years compared to 5.9% > 60 years) [19]. Our results may be used to emphasize the need for vaccination among younger individuals in certain risk groups to avoid severe course of disease.

In a clinical setting it is of great importance to recognize signs of a progressive disease and complications that are associated with a worse outcome. It is crucial both in terms of intensified treatment and to be able to prevent these to occur in the first place. Our findings that a previously diagnosed VTE (> 30 days prior to ICU admission) affect 90-day mortality is, to the best of our knowledge, a novel finding. Previous studies have suggested and identified an elevated risk for thromboembolic events to occur in COVID-19 patients and a strong relationship between present thromboembolic events and mortality in these severe cases of COVID-19 [21, 22]. However, we have not encountered studies investigating an earlier VTE diagnose and its impact on severe COVID-19 and VTE was not included as a risk factor for COVID-19-related death in a large study of over 17 million British e-health records[23]. We suggest awareness of previous thromboembolic events as well as prevention of new events. Our findings also imply that once in the ICU women are at equal risk of death as men.

## Strengths and limitations

Sweden has a number of high quality national health registries, including the Swedish ICU registry which has an overall coverage of ICU admission of 95% [13]. Also, health care is tax funded and available equally to all residents which reduces the risk of selection bias for non-medical reasons.

However, there are several limitations of this study including the risk of residual confounding. The strategies for timing of intubation may have changed throughout the study period and in some contexts a smaller proportion of patients may have been intubated at an early stage during wave two and three compared to the first wave. Patients admitted in the last couple of months of the study period may have received one or two doses of vaccine. Further, we also lack data on smoking habits and alcohol intake and that is, therefore, not assessed in this population. In addition, in this study we had no possibility to compare or match our cases with COVID-19 patients admitted to hospital but not in need of ICU care.

## Conclusion

Chronic kidney disease, type 2 diabetes, hypertension, rheumatoid arthritis, obesity, heart failure, and asthma showed the strongest associations with severe COVID-19 in young individuals (< 50 years of age) compared to old. For the young age-group, we also showed that sex differences exist, where type 2 diabetes and hypertension conferred a significantly higher relative risk for severe COVID-19 in women. After ICU admission, previous thromboembolism, chronic kidney disease, and type 2 diabetes were the most important risk factors for 90-day mortality among the young patients. Risk associations for co-morbidities were generally stronger among young persons compared to older and in women compared to men.

## Supplementary Information

**Additional file 1**: Registries and data bases used. Definition of co-morbidities classified by ICD-10. Flowchart for inclusion and exclusion. Associations of co-morbidities and 90-day mortalityin women. Associations of co-morbidities and 90-day mortalityin men. Significant associations of co-morbidities and 90-day mortalityin men and women.

## Data Availability

The datasets used and analyzed during the current study are available from the corresponding author on reasonable request.

## Abbreviations

AF: Atrial fibrillation
AOR: Adjuster Odds Ratio
ATC: Anatomical Therapeutic Chemical Classification
BMI: Body Mass Index
CI: Confidence Interval
COPD: Chronic Obstructive Pulmonary Disease
DM1: Diabetes Mellitus type 1
DM2: Diabetes Mellitus type 2
HF: Heart Failure
HT: Hypertension
IBD: Inflammatory Bowel Disease
ICD: International Classification of Diseases
ICU: Intensive care unit
IMV: Invasive mechanical ventilation
IQR: Interquartile Range
MI: Myocardial Infarction
OR: Odds ratio
RA: Rheumatoid Arthritis
SAPS III: Simplified Acute Physiology Score III
SD: Standard deviation
SID: Systemic Inflammatory Disease
SIR: Swedish Intensive care Registry
VTE: Venous Thromboembolism
