# Supplementary material for: Risk factors for severe COVID-19 in the young—before and after ICU admission
Source: Ann Intensive Care. 2023 Apr 25;13:31. doi: 10.1186/s13613-023-01127-8 (PMC10127156; doi:10.1186/s13613-023-01127-8)

# Supplementary

#### Suppl. 1. Registries and data bases used

- The Swedish Intensive Care Registry (SIR), governed by National Board of Health and Welfare
- Swedish National Patient Registry (NPR), governed by National Board of Health and Welfare
- The Swedish Prescribed Drugs Register, governed by National Board of Health and Welfare
- The Swedish Longitudinal Integrated Database for Health Insurance and Labour Market Studies (LISA), governed by Statistics Sweden

#### Suppl. 2. Definition of co-morbidities classified by ICD-10.

Diabetes mellitus type 2 (ICD E11 or prescribed antidiabetic drugs), diabetes mellitus type 1 (ICD E10), obesity (ICD E66), hyperlipidaemia (ICD E78 or prescribed lipid lowering drugs), chronic obstructive pulmonary disease (ICD J44), venous thromboembolism (ICD I26, I80), chronic kidney disease (ICD N18), heart failure (ICD I50.1, I50.9), atrial fibrillation (ICD I48), asthma (ICD J45), malignancy (ICD, D40-D48), rheumatoid arthritis (ICD M05, M06), inflammatory bowel disease (ICD K50, K51) and systemic inflammatory disease (ICD M30-M36).

**Hypertension definition**

Hypertension (ICD I10). Patients who picked-up prescribed anti-hypertension drugs within the preceding 12 months of index date were defined as having hypertension. Since beta-blockers, ACE-inhibitors, and Angiotensin receptor blockers may be prescribed for other diagnoses than hypertension, patients were not classified as having hypertension if these drugs were found in combinations with a diagnose of atrial fibrillation, myocardial infarction, heart failure and angina. An existing record of hypertension (I109) was superior to the pick-ups of prescribed drugs.

**Obesity definition**

In the analysis of risk factors for severe Covid-19, where cases are compared to controls, we only used the diagnose obesity (ICD E66). When analyzing the cases regarding 90-day mortality we used Body Mass Index (BMI) in addition to ICD code to set the obesity diagnose. This could be done since all patients admitted to the ICU had a verified BMI reported to SIR.

#### Suppl. 3. Flow chart for inclusion and exclusion.


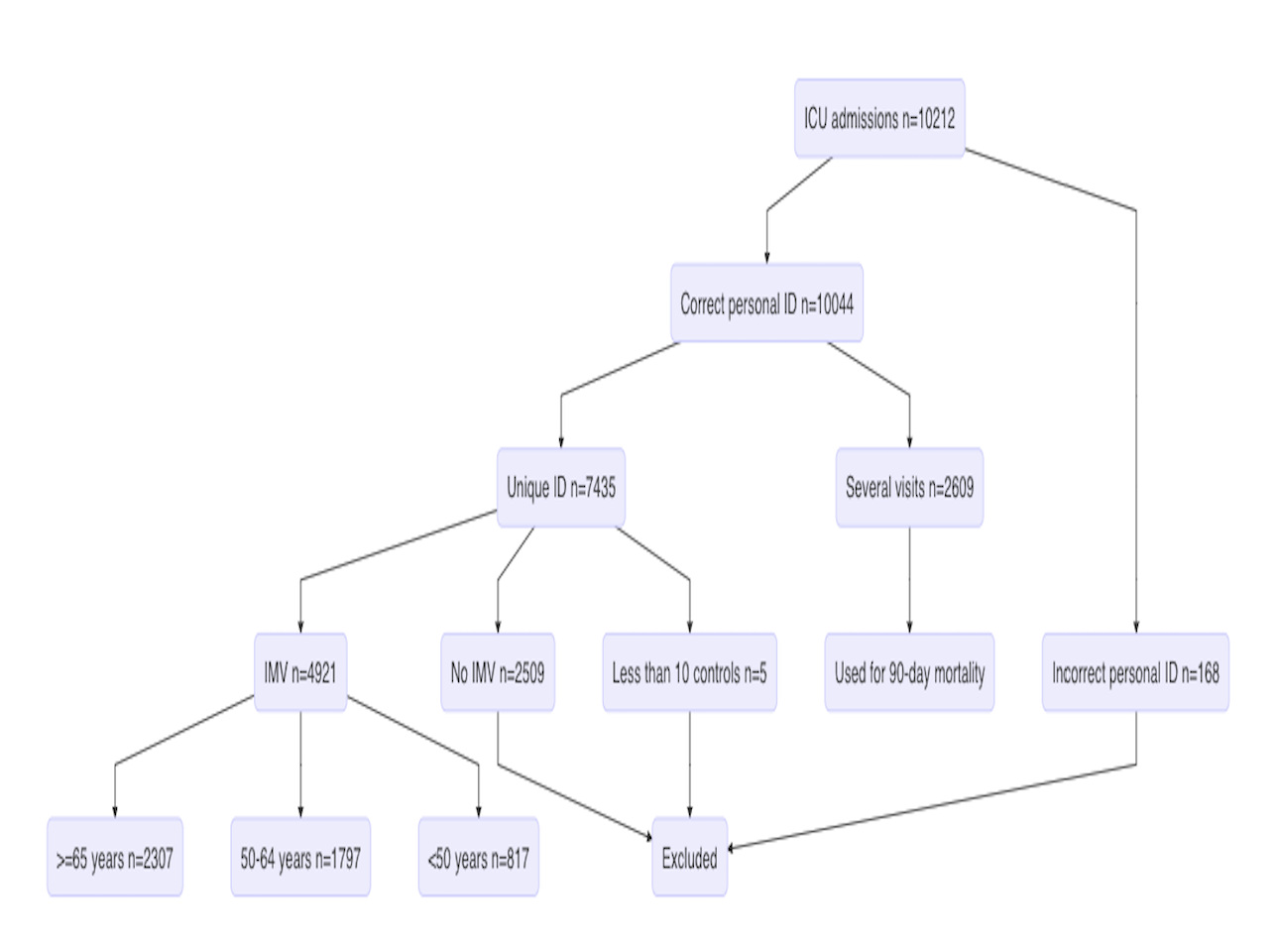


#### Suppl. 4a. Associations of co-morbidities and 90-day mortality (adjusted ORs with 95% CIs) in women.

###
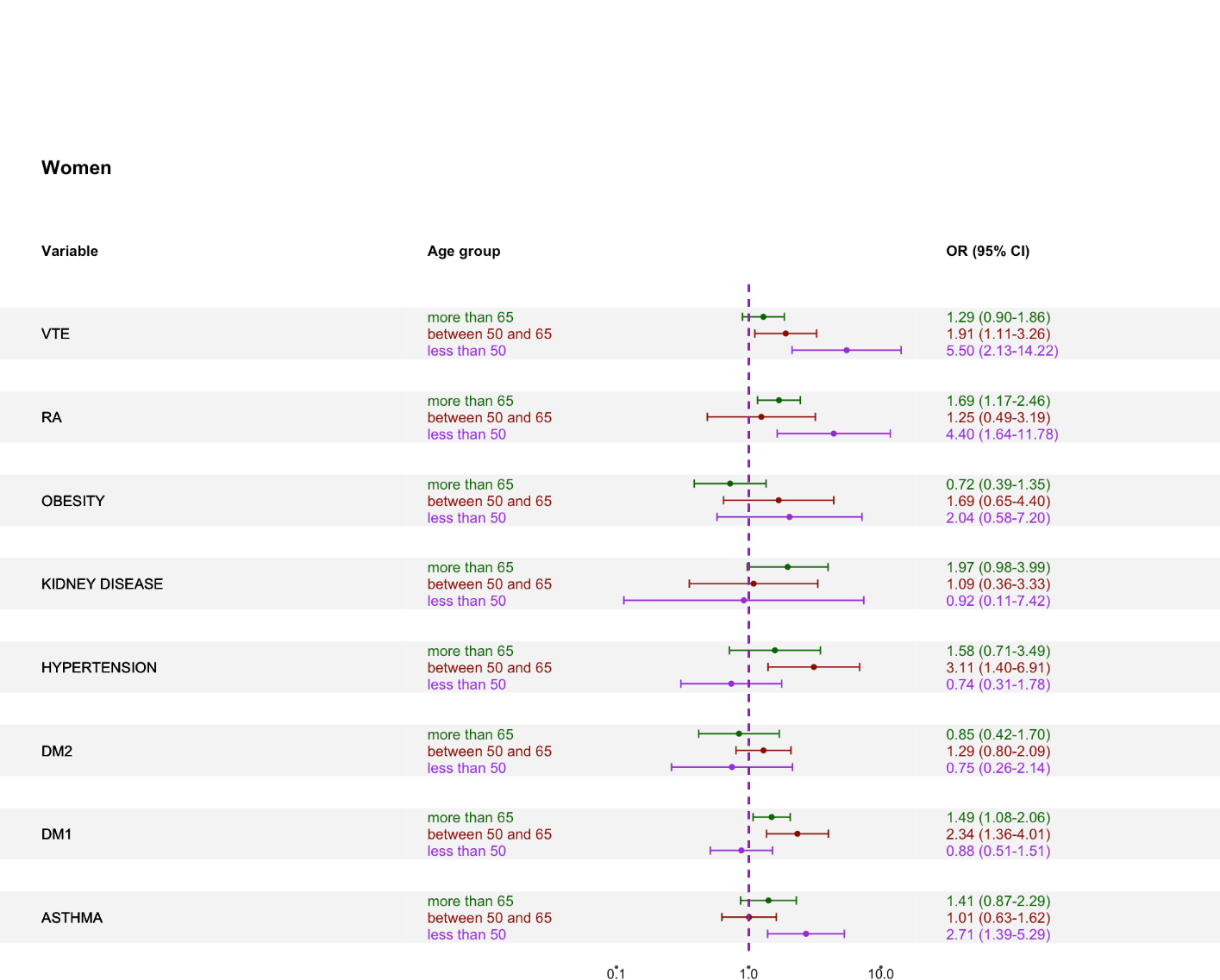


#### Suppl. 4b. Associations of co-morbidities and 90-day mortality (adjusted ORs with 95% CIs) in men.

###
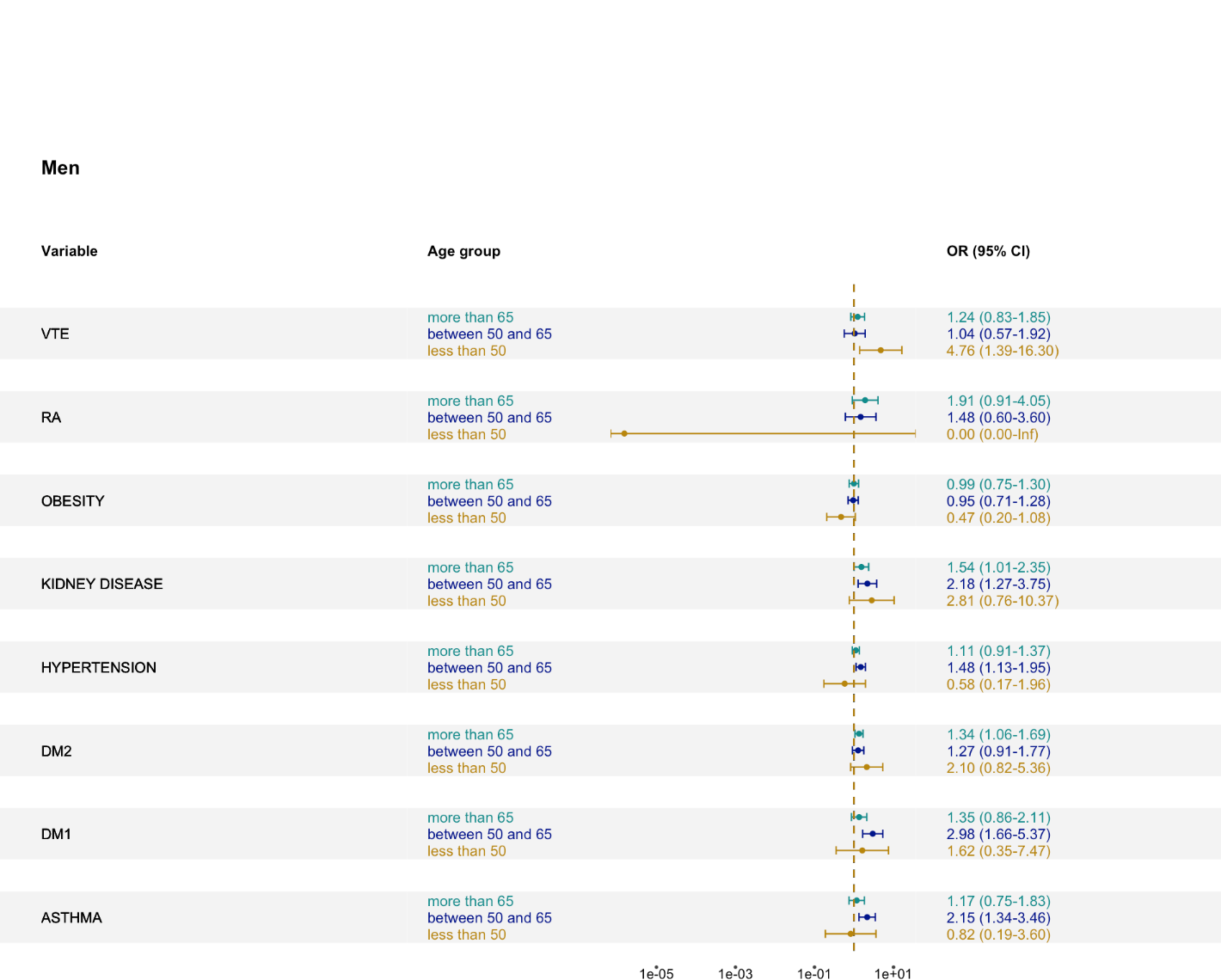


#### Suppl. 4c. Significant associations of co-morbidities and 90-day mortality (adjusted ORs with 95% CIs) in men and women.

#### Suppl. 5. Correlation of co-morbidities among all cases (n=4921) and how they cluster.


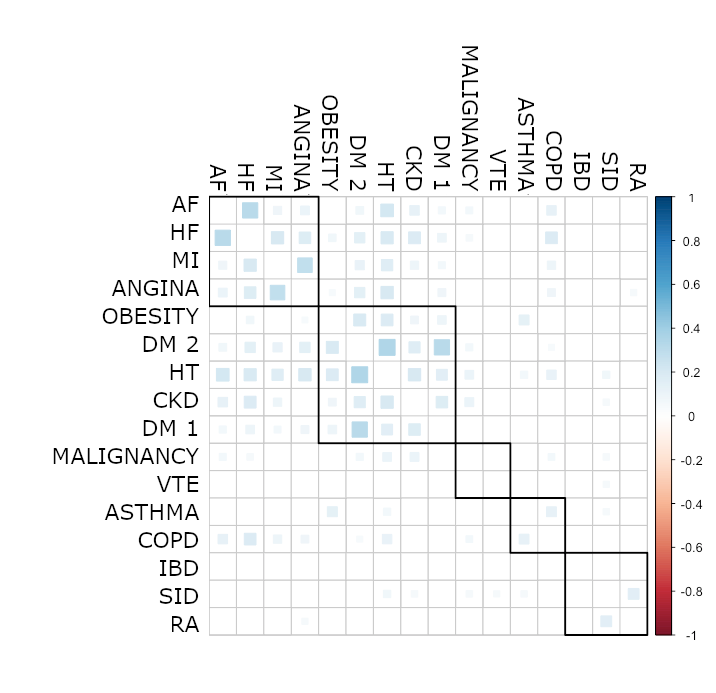

Supplement: Supplementary file 1 — Additional file 1: Registries and data bases used. Definition of co-morbidities classified by ICD-10. Flowchart for inclusion and exclusion. Associations of co-morbidities and 90-day mortalityin women. Associations of co-morbidities and 90-day mortalityin men. Significant associations of co-morbidities and 90-day mortalityin men and women. [file 13613_2023_1127_MOESM1_ESM.docx]
